# Absorption Patterns of Fucoidan Oligosaccharides from *Kjellmaniella crassifolia* in the Caco-2 Monolayer Cell Model and Their Pharmacokinetics in Mice

**DOI:** 10.3390/foods14091486

**Published:** 2025-04-24

**Authors:** Zhiying Xu, Qing Xia, Liu Li, Yuxin Shi, Yuan Gao, Yichao Ma, Shu Liu, Yunhai He, Qiukuan Wang, Dandan Ren

**Affiliations:** 1 College of Food Science and Engineering, Dalian Ocean University, Dalian116023, China; 2 Xinjiang Standard Inspection Product Testing and Certification Co., Ltd., Urumqi 830000, China; 3 National R&D Branch Center for Seaweed Processing, Dalian 116023, China; 4 Key Laboratory of Aquatic Product Processing and Utilization of Liaoning Province, Dalian 116023, China

**Keywords:** fucoidan oligosaccharides, structural analysis, column separation, bidirectional transmembrane transport

## Abstract

Oligosaccharides possess characteristics such as low molecular weight, good solubility, and high bioavailability, which make them better absorbed than fucoidan. This study hypothesizes that fucoidan oligosaccharides can be absorbed by intestinal epithelial cells and quickly enter the bloodstream, with a rapid absorption rate. In this study, fucoidan oligosaccharides were obtained through acid degradation and Bio Gel column separation. By analyzing the chemical composition and molecular weight, oligosaccharides with smaller molecular weights and simpler monosaccharide compositions were selected for further research. A cell model and pharmacokinetic studies in mice were established to analyze the absorption patterns of the oligosaccharides. The results showed that after acid degradation and column separation, high-molecular-weight oligosaccharides SPF1 with a molecular weight range of 1.63 × 10^4^ to 2.14 × 10^5^ Da and the low-molecular-weight oligosaccharides SPF2 with a molecular weight range of 244.22 to 1545.36 Da were obtained. In cell transport and uptake experiments, the transport of SPF1 and SPF2 was positively correlated with time and negatively correlated with concentration. The transport rates of SPF1 and SPF2 ranged from 20% to 70%, with *P_app_* values greater than 1 × 10^−5^ cm/s. In the pharmacokinetics study, the blood concentration of the oligosaccharides in mice was simulated and analyzed using DAS 2.0, which indicated that the fucoidan oligosaccharides exhibited good absorption characteristics in vivo and in vitro. Therefore, fucoidan oligosaccharides with smaller molecular weights are more easily absorbed, which provides a theoretical basis for the application of fucoidan oligosaccharides.

## 1. Introduction

Fucoidan is a potential natural product primarily composed of sulfate groups and L-fucose [1], which is commonly found in brown algae and echinoderms [2]. Its chemical structure consists of α- (1→3)-L-fucopyranose residues or alternating α- (1→3)-linked and α- (1→4)-linked L-fucopyranose or α- (1→2)-L-fucopyranose residues [3,4,5]. Fucoidan exhibits various biological activities, which include anticoagulant, antioxidant, anti-inflammatory, and antitumor effects, immune regulation, etc. [6,7]. Current research indicates that fucoidan has a high molecular weight and viscosity, which results in poor solubility in solution and affects its medicinal efficacy [8,9]. Additionally, it has low bioavailability and leads to the use of fucoidan oligosaccharides in functional foods and pharmaceuticals.

Fucoidan oligosaccharides are degradation products that are generated from fucoidan through methods such as mild acid hydrolysis, enzymatic hydrolysis, or free radical depolymerization. They have a smaller molecular weight to absorb and utilize easier for the intestine. Fucoidan oligosaccharides possess antioxidant, anticancer, and antiviral activities and prebiotic properties. Oligosaccharides can compensate for the shortcomings of fucoidan, such as its high molecular weight and low bioavailability [10].

With the development of technology, research on the activity of fucoidan has become increasingly extensive. Nagamine et al. found that fucoidan could be absorbed in the small intestine [11]. Kizuku K et al. discovered that fucoidan was excreted in urine after intestinal digestion [12]. Additionally, it was found through E. Z’s cell model and Zhao’s experimental animal model that low-molecular-weight fucoidan was more conducive to absorption [13,14]. However, studies on the in vitro absorption and in vivo pharmacokinetics of these oligosaccharides remain limited.

In this study, the fucoidan extracted from *Kjellmaniella crassifolia* was used as the raw material, and fucoidan components named SPF1 and SPF2 were obtained through acid degradation and column separation. A Caco-2 monolayer cell model was established to investigate the in vitro transport and uptake of F, SPF1, and SPF2. Additionally, fluorescein isothiocyanate (FITC) was used to fluorescently label these substances, which was followed by in vivo pharmacokinetic experiments in mice. This research worked not only to explore the absorption and metabolic patterns of brown algae oligosaccharides but also to provide a theoretical basis for their application.

## 2. Materials and Methods

### 2.1. Materials and Reagents

Fucoidan from *Kjellmaniella crassifolia*, named “F” (National R&D Branch Center for Seaweed Processing of Dalian Ocean University, Dalian, China). Caco-2 cell (Shanghai Institute of Life Sciences, Chinese Academy of Sciences, Shanghai, China). DMEM (high sugar), non-essential amino acids, 0.25% trypsin-EDTA, penicillin-streptomycin mixture, Hanks, CCK-8 kit, and AKP/ALP activity detection kit (Beijing Soleibao Technology Ltd., Beijing, China).Bio-Gel P10 (American Bio-Rad Corporation, Hercules, CA, USA).

### 2.2. Instruments

RE1600 Epithelial Cell Voltage Resistance Meter (Beijing Jinghong Hongtai Technology Co., Ltd., Beijing, China). TS100F Inverted Microscope (Nikon Co., Ltd. of Japan, Tokyo, Japan). 1260 High-Performance Liquid Chromatograph (Agilent Technologies, Inc., Santa Clara, CA, USA) IS50 Infrared Spectrometer (Thermo NICOLET, Waltham, MA). UV-9000S Ultraviolet-Visible Spectrophotometer (Shanghai Bajiu Industrial Co., Ltd., Shanghai, China). TGL2050 High-Speed Frozen Centrifuge (Guohua Electric Co., Ltd., Changzhou, China) BT100-2J Peristaltic Pump (Shanghai Hu Xi Analytical Instrument Factory Co., Ltd., Shanghai, China). SBS-160 Receiver (Shanghai Huxi Analytical Instrument Factory Co., Ltd., Shanghai, China). Synergy H Enzyme Labeling Instrument (BioTek Instruments, Inc., Winooski, VT, USA). SCIENTZ-10N Vacuum Freeze Dryer (Ningbo Xinzhi Biotechnology Co., Ltd., Ningbo, China). 371 CO_2_ Incubator (Thermo Fisher Scientific Inc., Waltham, MA, USA). SCIENTZ-IID Ultrasonic Cell Crusher (Ningbo Xinzhi Biotechnology Co., Ltd., Ningbo, China). Synergy H/HM Multifunctional Microplate Reader (BioTek Instruments, Inc., Winooski, VT, USA).

### 2.3. Preparation of Fucoidan Oligosaccharides

The fucoidan was prepared by acid hydrolysis [3], then an equal volume of ethanol was added and left standing overnight. The solution was centrifuged at 4000 rpm for 10 min to obtain the supernatant. The ethanol in the supernatant was removed by rotary evaporation and then lyophilized and named SF. SF was separated by the Bio-Gel P10 (1.6 cm × 100 cm), the mobile phase was 0.5 mol/L NH_4_HCO_3_, the flow rate was 0.15 mL/min, and each tube was 10 min. The total sugar content was determined by the phenol-sulfuric acid method, and the elution curve was drawn. The corresponding components were collected according to the elution curve. Salts were removed by repeated rotary evaporation, then lyophilized. The separated components were named SPF1 and SPF2 according to the order of collection.

### 2.4. Chemical Constituents of F, SPF1, and SPF2

The total sugar content was determined using the phenol-sulfuric acid method [15]. The sulfate group content was measured by the barium sulfate turbidity method [16]. The content of uronic acids was determined using the carbazole colorimetric method [17]. The monosaccharide composition was analyzed using pre-column derivatization high-performance liquid chromatography (HPLC) [18]. The distribution of the relative molecular weight of SPF1 was measured using high-performance gel permeation chromatography combined with multi-angle laser light scattering (HPGPC-MALLS) [19]. The distribution of the relative molecular weight of SPF2 was determined using mass spectrometry.

The chemical structure of the oligosaccharides was analyzed using infrared spectroscopy [20]. The potassium bromide (KBr) pellet method involved accurately weighing 2 mg of the sample and 100 mg of potassium bromide, then drying them in an oven to constant weight, which was ground into a powder and pressed into pellets. A Fourier-transform infrared spectrometer was used to scan the wavelength range of 4000–400 cm^−1^, with 100 mg of potassium bromide as the background. Spectroscopic analysis was performed, with three parallels for each sample.

### 2.5. Caco-2 Cytotoxicity Assay

Caco-2 cells were seeded in a 96-well culture plate at 1 × 10^5^ cells/mL. 100 μL of cell suspension was added to each well, and 200 μL of PBS solution was added to the periphery of the 96-well plate. The Caco-2 cells were randomly divided into a blank group (no cells added, only DMEM medium), a control group (cells, DMEM medium, no sugar solution), and an experimental group (the final concentration of the sample was 37.5, 75, 150, 300, and 600 μg/mL). Three replicate wells in each group were added with 10 μL of CCK-8 reagent to each well and continued to incubate for 3 h after culturing for 24 h at 37 °C in an atmosphere of 5% CO_2_. The OD value at a wavelength of 450 nm was measured by a standard instrument.

### 2.6. Establishment of Caco-2 Cell Monolayer Model

Caco-2 cells (7 × 10^4^/cm^2^) cultured to log phase were seeded in Transwell (0.4 μm, 1.12 cm^2^) chambers. Then, the cells were inoculated. First, the cells in the culture flask were digested with trypsin and blown into a cell suspension. Then, 0.4 mL of the cell suspension was added to the upper chamber (apical side, AP), and 0.6 mL of complete culture medium was added to the lower chamber (basolateral side, BL). The cells were cultured at 37 °C with 5% CO_2_, with the culture medium changed every two days during the first week and daily in the following two weeks. The success of the model was validated by measuring transepithelial electrical resistance (TEER), alkaline phosphatase (AKP) activity, and cell permeability indicators [21].

### 2.7. Transport and Uptake Experiments

The bidirectional transmembrane transport experiment was carried out with the Caco-2 cell monolayer formed after 21 days of culture [22]. The cell culture medium on both sides of the Transwell chamber was discarded, and the cells were rinsed twice with Hanks buffer. The specific operations of the bidirectional transport experiment were as follows: the AP-BL method used Hanks buffer to prepare sample solutions of different concentrations (F: 75 µg/mL; SPF1 and SPF2: 37.5 µg/mL, 75 µg/mL, 150 µg/mL, and 300 µg/mL). The AP side added 0.5 mL of sample solution, and the BL side added 1.5 mL of Hanks buffer. At 0.5 h, 1 h, and 2 h, 20 µL was taken from the BL side, and the same volume of Hanks’ buffer was added to replenish it. The BL-AP method added 1.5 mL of sample solution to the BL side, and the AP side added 0.5 mL of Hanks buffer. After incubation for 0.5 h, 1 h, and 2 h, 20 µL was taken from the AP side, and the same volume of Hanks’ buffer was added to replenish it. The total sugar content of the solutions drawn in different periods was measured by the phenol-sulfuric acid method to compare the transport effects on both sides of the Caco-2 cell monolayer.

The uptake experiment could be performed together with the transport experiment. After the transport experiment, both sides of the cells were washed with Hanks buffer, and all cells were scraped off. The cells were sonicated at 20% power for 3 s, with a 10 s interval, repeated 10 times. The mixture was centrifuged at 8000 rpm for 10 min at 4 °C, and the supernatant was collected to determine the total sugar content.

### 2.8. Fluorescent Labeling of F and SPF2

Following the method of Ziming [23]. Moreover, 400 mg of F and SPF2 were added to 15 mL of 0.2 mol/L phosphate buffer (pH 8). Moreover, 400 mg of tyramine and 150 mg of sodium cyanoborohydride were added. The mixture was reacted on a shaker at 150 r/min and 37 °C for 96 h. After centrifugation, the supernatant was collected and mixed with 4 times its volume of absolute ethanol. The solution was subjected to alcohol precipitation and centrifugation, which yielded freeze-dried products F-Tyr and SPF2-Tyr. Furthermore, 200 mg of F-Tyr and SPF2-Tyr were added to 10 mL of 0.5 mol/L sodium bicarbonate solution, followed by the addition of 25 mg of FITC. The reaction was conducted in the dark at room temperature for 24 h. After centrifugation, the supernatant was collected and mixed with 4 times its volume of absolute ethanol, followed by repeated alcohol precipitation and centrifugation to obtain the precipitate, which was freeze-dried to yield T-F and T-SPF2.

Perform fluorescence verification on the fluorescently labeled F and SPF2, according to Liu’s protocol [24]. The samples were prepared at appropriate concentrations for UV-Vis spectroscopy (spectral range: 200–650 nm), fluorescence spectroscopy (excitation wavelength set at 490 nm, slit width set at 5 nm, emission wavelength scan range: 450–650 nm), and infrared spectroscopy (4000–400 cm⁻¹) to verify the fluorescence labeling.

### 2.9. T-F and T-SPF2 Quantitative Analysis

For this, 1 mg/mL T-F and T-SPF2 standard solutions were prepared with PBS buffer, and the standard solutions were diluted to create T-F and T-SPF2 standard series solutions at concentrations of 5, 12.5, 25, 50, and 125 μg/mL. Then, 80 μL of blank plasma was added to 20 μL of different concentrations of T-F and T-SPF2 standard series solutions to prepare T-F and T-SPF2 solutions (1, 2.5, 5, 10, and 25 μg/mL). The blank sample was of the same volume as the PBS buffer, and the fluorescence intensity was measured (λex = 490 nm and λem = 530 nm).

According to the above method, samples were prepared at high (T-F: 25 μg/mL, T-SPF2: 25 μg/mL), medium (T-F: 5 μg/mL, T-SPF2: 5 μg/mL), and low (T-F: 1 μg/mL, T-SPF2: 1 μg/mL) concentrations. The solution was measured for fluorescence intensity to calculate the recovery rate, precision, and stability of the method.

(1) $$\mathrm{Recovery}\;\mathrm{rate}\;\left(\%\right)=\frac{\mathrm{Concentration}\;\mathrm{of}\;\mathrm{the}\;\mathrm{drug}\;\mathrm{after}\;\mathrm{regression}}{\mathrm{Actual}\;\mathrm{concentration}\;\mathrm{of}\;\mathrm{the}\;\mathrm{prepared}\;\mathrm{drug}}\times 100$$

### 2.10. Metabolic Kinetics Study in Mice

Twenty male Kunming mice (24–28 g) were divided into the following three groups: blank, T-F, and T-SPF2, with 5 mice in each group. After adapting to the environment for 3 days, the experiment was conducted. The mice were fasted for 12 h before administration but allowed to drink water freely. T-F and T-SPF2 were prepared with saline for oral gavage, with a dosage of 0.067 mg/kg and a volume of 1 mL/100 g. Blood samples of 200 μL were collected from the tail at different time points (0.5, 1, 2, 4, 6, 8, 12, 24, and 48 h) into EDTA-treated tubes. The samples were centrifuged to obtain plasma. The fluorescence intensity was measured according to the method described in Section 2.9, and the data were analyzed using DAS 2.0.

### 2.11. Statistical Analysis

Each experiment had three parallel data, and the results were expressed as mean ± standard deviation. SPSS 17.0 was used to conduct variance analysis on the data. *p* < 0.05 was considered a significant difference, and *p* < 0.01 was considered an extremely significant difference.

## 3. Results

### 3.1. Preparation of SPF1 and SPF2

**Figure 1:**
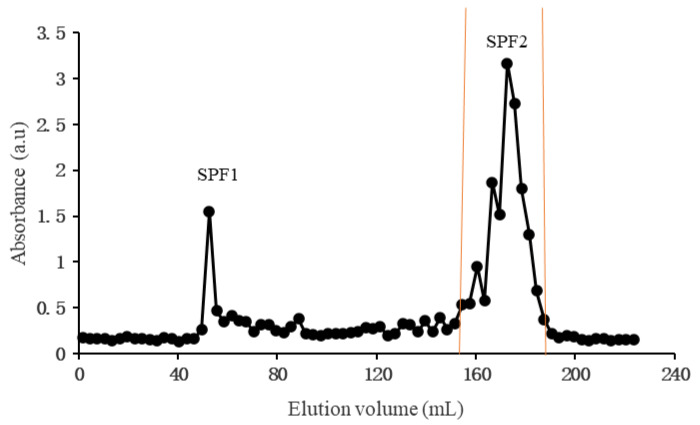
Bio Gel P10 elution curves of SPF1 and SPF2.

The low-molecular-weight fucoidan obtained by acid degradation was separated and purified by Bio Gel P10 to obtain an elution curve (Figure 1). The SPF1 and SPF2 were collected according to the elution curve, and their yields were 3.73% and 69.73%, respectively.

### 3.2. Molecular Characterization of F, SPF1, and SPF2

#### 3.2.1. Chemical Composition of F, SPF1, and SPF2

According to the phenol-sulfuric acid method, barium sulfate turbidity method, and carbazole colorimetric method. The total sugar content of F, SPF1, and SPF2 was 51.90%, 68.67%, and 59.70%, respectively. The sulfate group content was 25.58%, 15.33%, and 16.89%, respectively. While the uronic acid content of SPF1 was 2.15%, no uronic acid was detected in F and SPF2. According to the HPGPC-MALLS method and liquid chromatography-mass spectrometry, the molecular weight range of SPF1 was determined to be 1.63 × 10^4^~2.14 × 10^5^ Da. The molecular weight range of SPF2 was found to be 244.22~1545.36 Da. After column separation, the total sugar content of the two components significantly increased, with the total sugar content of the SPF1 component being significantly higher than that of the crude sugar F and SPF2. Additionally, the sulfate group content of the oligosaccharides significantly decreased after degradation. Generally, under acidic degradation conditions, branched glycosidic bonds were more susceptible to hydrolysis than linear glycosidic bonds [25]. The glycosidic bonds connected to uronic acids were more resistant to hydrolysis, while the non-reducing ends were more likely to undergo hydrolysis. During hydrolysis, there was a selective loss of sulfate substituents. Thus, the decrease in sulfate group content in the degraded oligosaccharides was observed. The uronic acid content in SPF1 increased significantly after column separation, while SPF2 showed no detectable uronic acid. These results indicated that SPF2 was purer compared to SPF1, with lower content of other monosaccharides.

High-performance liquid chromatography (HPLC) was used to analyze the monosaccharide components of algal polysaccharides. The results are shown in Table 1. The algal polysaccharide was primarily composed of sulfated fucose, along with small amounts of xylose, rhamnose, mannose, galactose [26], and glucuronic acid [27]. Both SPF1 and SPF2 contained fucose, mannose, rhamnose, glucuronic acid, galactose, glucose, and xylose, but the content varied significantly. SPF1 was mainly composed of mannose, glucuronic acid, galactose, and rhamnose. The mannose content was more than three times that of fucose, and glucuronic acid was twice that of fucose. The data indicated that the high total sugar content and increased uronic acid content in SPF1 were due to the presence of a larger amount of high-molecular-weight heteropolysaccharides. The fucose content in SPF2 was significantly higher than that in F and SPF1, while the content of other monosaccharides was less than 1%. These indicated that SPF2 was primarily composed of fucose.

#### 3.2.2. Infrared Spectroscopy Analysis

**Figure 2:**
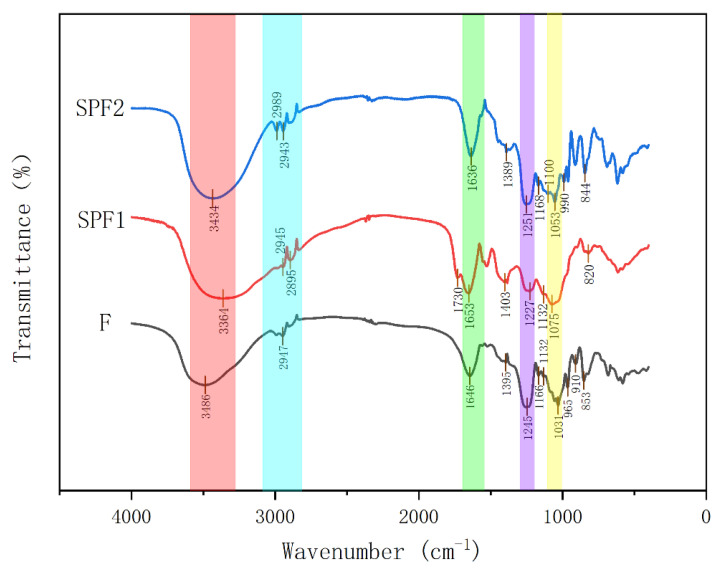
FT-IR spectra of F, SPF1, and SPF2.

The characteristic peaks of F, SPF1, and SPF2 are summarized in Figure 2. From the figure, it can be seen that F, SPF1, and SPF2 all contained an O-H vibration around 3400 cm^−1^, a C-H vibration around 2945 cm^−1^, a C=O vibration around 1650 cm^−1^, an S=O vibration around 1250 cm^−1^, and a C-O-C/C-OH stretching vibration peak around 1060 cm^−1^. All of these were characteristic peaks of sugars. The absorption peak at 1250 cm^−1^ for SPF1 and SPF2 was weaker compared to F, which was consistent with the results of sulfate group determination. At around 850 cm^−1^, F and SPF2 exhibited an absorption peak, while SPF1 showed an absorption peak around 820 cm^−1^. The data indicated that the sulfate groups in SPF1 were attached at the C2 and C3 positions, which represented sulfates connected by flat bonds. The sulfate groups in F and SPF2 were connected at the C4 position of fucose, which represented sulfates connected by upright bonds [28].

### 3.3. Cell Viability Assay

**Figure 3:**
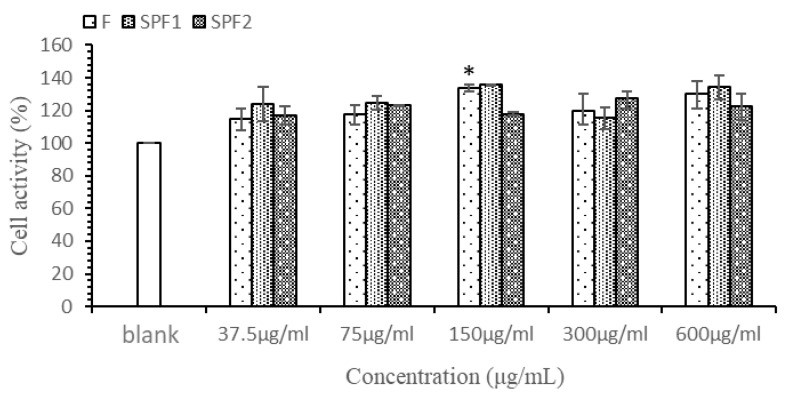
The cytotoxicity of F, SPF1, and SPF2 on Caco-2 cells (* *p* < 0.05).

The results of the effect of different concentrations of F, SPF1, and SPF2 on the viability of Caco-2 cells measured by the CCK-8 method are shown in Figure 3. The figure indicated that within the concentration range of 37.5–600 µg/mL, F, SPF1, and SPF2 exhibited no toxic effects on Caco-2 cells and had a certain promoting effect on cell growth. Both F and SPF1 showed an increase in cell viability with increasing concentration in the range of 37.5–150 µg/mL. Although there was a downward trend after 150 µg/mL, they still demonstrated a promoting effect compared to the blank control. SPF2 also had a promoting effect on cells within the concentration range of 37.5–600 µg/mL and could be used for subsequent transport experiments.

### 3.4. Establishment of the Cellular Intestinal Model

There was a good relationship between the integrity of the single-layer model and the TEER value. The general TEER value was between 200 and 1000 Ω·cm^2^ [29]. The value was greater than 200 Ω·cm^2^, indicating that the Caco-2 monolayer cell membrane was dense and intact. The larger the TEER value, the tighter the cell monolayer connection. The TEER value of the cells increased rapidly from the 7th day of culture until the TEER value of the cells stabilized on the 17th day, which reached about 310 Ω·cm^2^. The data showed that the cells grew slowly in the first week and began to divide rapidly in the second week. After 17 days, the TEER value tended to be stable, which indicated that cell differentiation was completed. At this time, the integrity of the monolayer membrane of Caco-2 cells was good, and the intercellular connections were tight.

**Figure 4:**
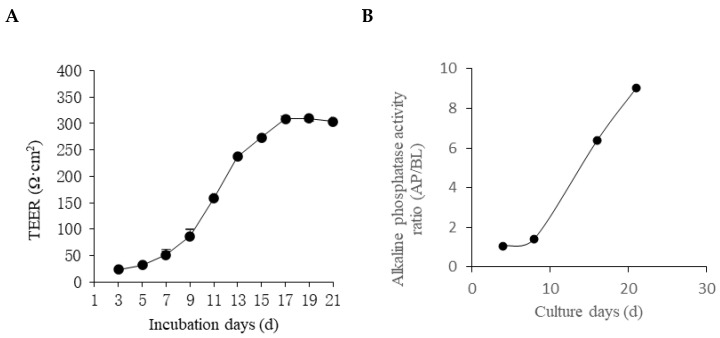
The changes in TEER (**A**) and alkaline phosphatase activity ratio (**B**) of the Caco-2 monolayer cell model with culture time.

Alkaline phosphatase was a representative enzyme among intestinal cell-specific enzymes secreted by Caco-2 cells. Alkaline phosphatase was a marker enzyme on the microvilli of small intestinal epithelial cells and could be used to measure functional activities such as the degree of differentiation, material absorption, and transport of intestinal epithelial cells [30]. The ratio of alkaline phosphatase on the 21 d of cell culture was about 9 times that of the 1 d. In Figure 4, the data indicated that the cells had differentiated into polarity, which had the carriers and enzymes required for absorption and transport for transport experiments [31].

Fluorescein was a water-soluble substance with the characteristics of membrane permeability, large molecular weight, and difficult metabolism. This substance was difficult to transport across cells and intercellular channels. It could be used as a marker to detect the permeability of the cell monolayer to assess the integrity of the cell monolayer [32]. Penetration testing was performed after 21 d of culture. According to the formula calculation, the apparent permeability coefficient of fluorescein was 4.16 × 10^−7^ cm/s, which was within the range of 0.1~0.7 × 10^−6^ cm/s specified in the permeation experiment [33]. The data indicated that the Caco-2 cell monolayer membrane exhibited good permeability.

In summary, Caco-2 cells already possessed the enzymes and carriers required for transport. The monolayer cell membrane brought by tight junctions between cells has good integrity and good permeability. These findings indicated that the modeling of the Caco-2 cell monolayer model was successful. The model could be used as an in vitro cellular model to simulate intestinal epithelial uptake for subsequent transport and uptake experiments.

### 3.5. Transport and Uptake Experiments

#### 3.5.1. Transport Experiment

**Figure 5:**
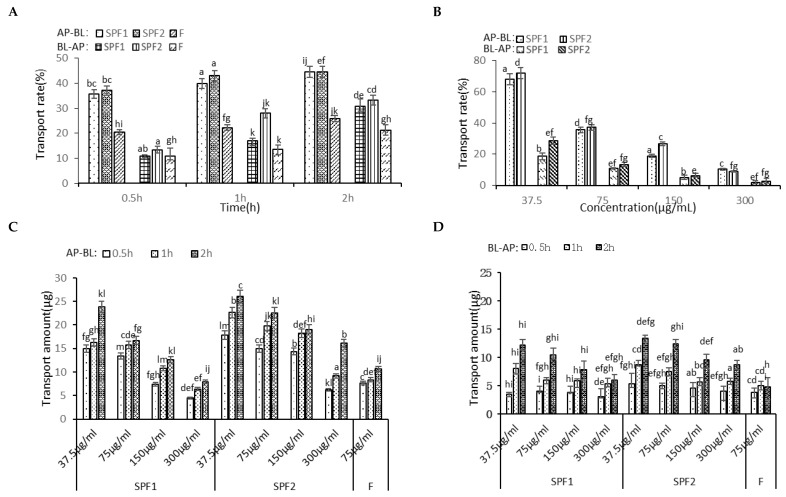

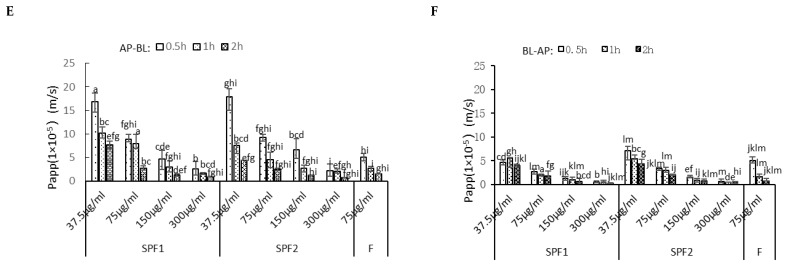
The effects of different time points and concentrations of fucoidan oligosaccharides on transport across both sides of the Caco-2 monolayer cell model (**A**,**B**), as well as the transport amounts and apparent permeability coefficients (**C**–**F**). Differences with different letters in the same row are significant (*p* < 0.05), while those with the same letter are not significant (*p* > 0.05). AP-BL, apical to basolateral; BL-AP, basolateral to apical.

As shown in Figure 5A, the effects of different time points on the transport of F, SPF1, and SPF2 from the AP-BL (apical to basolateral) side and the BL-AP (basolateral to apical) side were observed with a fixed concentration of 75 μg/mL. With increasing time, the transport rates on both sides increased to varying degrees. The transport amounts of SPF1 and SPF2 continuously increased without reaching saturation. These findings indicated a time-dependent relationship [34]. Figure 5B illustrated the impact of different concentrations of SPF1 and SPF2 on cell transport. With a fixed time of 0.5 h, as the concentration increased, the transport rates decreased. This phenomenon suggested that the transport of fucoidan and its oligosaccharides relied on specific carriers. The decrease in transport rates might be due to the limited number of carriers [29], and lower concentrations were more favorable for drug absorption [35].

Comparing the transport amounts, it was found that the smaller molecular weight SPF2 was more easily transported, while the larger molecular weight F had a significantly lower transport rate than SPF1 and SPF2. This finding showed that smaller molecular weights corresponded to better absorption efficiency [36]. However, the transport amount of F on the BL side tended to reach saturation as time increased. In the same time period, the transport amounts consistently decreased with increasing concentrations, and the transport amount on the BL side was less than that on the AP side. This finding indicated that the transport capacity on the AP side was higher than that on the BL side, with absorption being greater than efflux [37]. The apparent permeability parameters (*P_app_*) reflected the absorption capacity of substances in the intestine. According to international criteria, a transport rate of 70–100% and *P_app_* value > 1 × 10^−5^ cm/s indicated good absorption, a transport rate of 20–70% and *P_app_* value between 1.0 × 10^−6^ and 1.0 × 10^−5^ cm/s indicated moderate absorption, and a transport rate of 0–20% and *P_app_* value < 1.0 × 10^−6^ cm/s indicated poor absorption [38]. The *P_app_* value of F was close to 1 × 10^−5^ cm/s, but its transport rate was around 20%, resembling poorly absorbed substances. The transport rates of SPF1 and SPF2 were between 20 and 70%, with *P_app_* value > 1 × 10^−5^ cm/s. These results significantly indicated that SPF1 and SPF2 were classified as moderately absorbable substances that could be effectively absorbed in the intestine.

#### 3.5.2. Uptake Experiments

**Figure 6:**
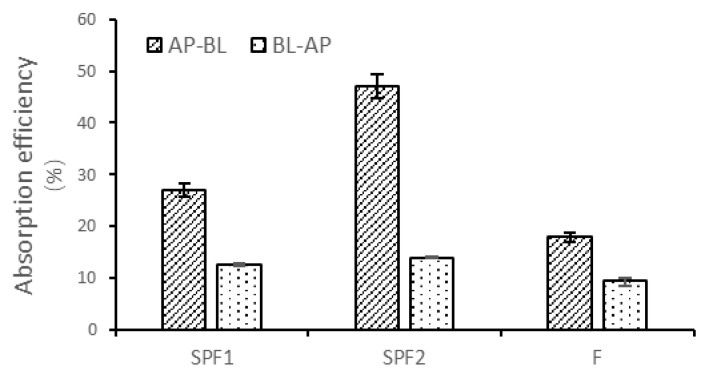
Absorption rates of F, SPF1, and SPF2 on both sides of Caco-2 cells at the same concentration. AP-BL, apical to basolateral; BL-AP, basolateral to apical.

After the cells were transported, the intracellular sugar content was measured by cell lysis. In the Figure 6, SPF2 showed the highest absorption rate, with 47.07% on the AP side and 13.96% on the BL side. This was followed by SPF1 and F, with absorption rates of 26.97% and 17.96% on the AP side and 12.55% and 8.64% on the BL side. This indicated that smaller molecular weight substances were more easily absorbed by the cells. The absorption was more efficient on the AP side compared to the BL side.

### 3.6. Fluorescent Labeling of F and SPF2

#### 3.6.1. Degree of Substitution

**Figure 7:**
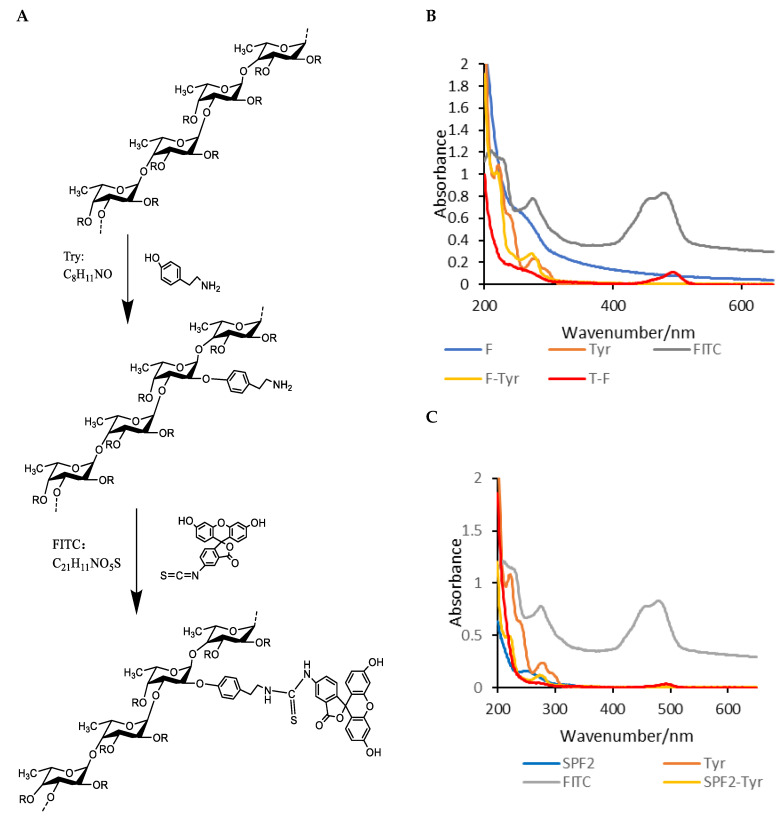
The fluorescence labelling process (**A**) and UV-Vis spectra of T-F and T-SPF2 (**B**,**C**).

By measuring the fluorescence intensity of T-F and T-SPF2 and substituting these values into the regression equation, the fluorescence substitution rates were obtained as 0.030% and 0.021%, respectively. The oligosaccharide fluorescent markers obtained using Tyr as a linker arm have a slightly lower substitution rate compared to polysaccharides. The purified SPF2 consisted of a series of low-molecular-weight sulfated fucose or fucoidan oligosaccharides and contained some fucoidan monosaccharides. The number of reducing ends was lower than that of polysaccharides, and the molecular weight was smaller. During the alcohol precipitation, T-SPF2 was lost due to incomplete precipitation. Under acidic conditions, the proportion of open hemiacetal groups at the reducing ends of fucoidan oligosaccharides was relatively high, which made it easier for them to react with the amino groups of Tyr. The polysaccharides were converted into amino polysaccharides, which could be labeled by reacting with FITC. The synthesis process and chemical structures are illustrated in Figure 7A.

#### 3.6.2. UV-Vis Spectrum

As shown in Figure 7B,C, F-Tyr and SPF2-Tyr exhibited a significant absorption peak at 274 nm, which is also present in Tyr. T-F and T-SPF2 showed a notable absorption peak at 493 nm, which was consistent with the characteristic peak of FITC. The absorption intensity of T-F was significantly higher than that of T-SPF2, which was consistent with the results of the fluorescence substitution degree. These findings indicated that F and SPF2 successfully bound with Tyr, and the productions T-F and T-SPF2 were successfully formed through binding with FITC. Furthermore, at the same concentration, the FITC content in T-F was higher than that in T-SPF2.

#### 3.6.3. Fluorescence Spectrum

**Figure 8:**
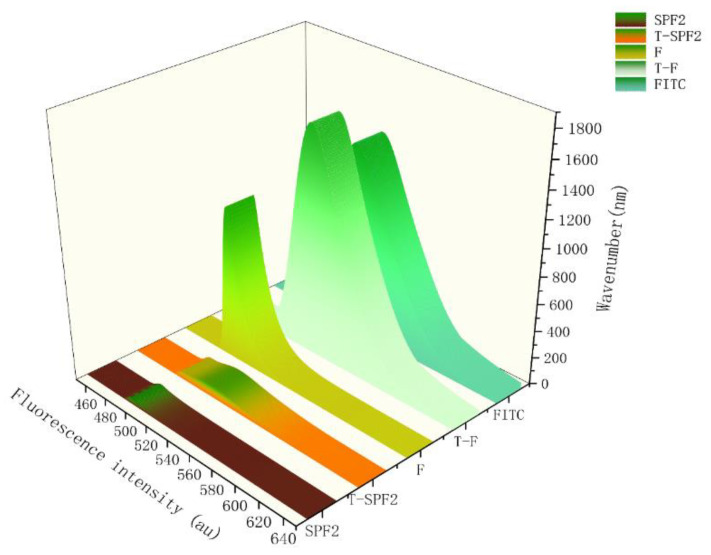
Fluorescence spectra of F, SPF2, FITC, T-F, and T-SPF2.

FITC exhibited a prominent absorption peak at 527.5 nm. T-F and T-SPF2 showed significant absorption peaks at 529.5 nm and 524 nm in the Figure 8, which were close to the chromatographic peak of FITC. This finding indicated that both F and SPF2 labeled with Tyr as a linker were successfully connected with FITC [39], and there were phenomena of blue shift and red shift.

#### 3.6.4. Fourier-Transform Infrared Spectroscopy

**Figure 9:**
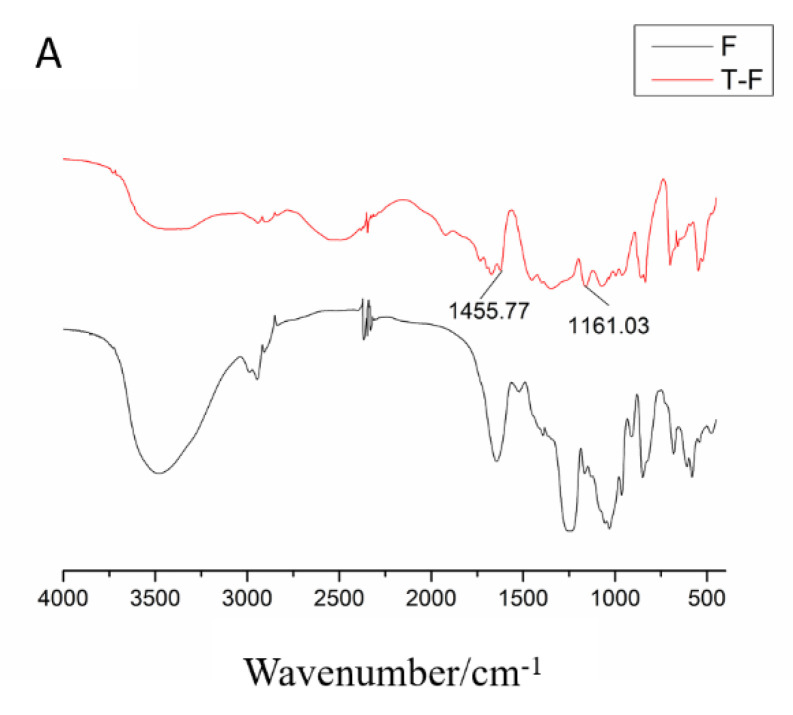

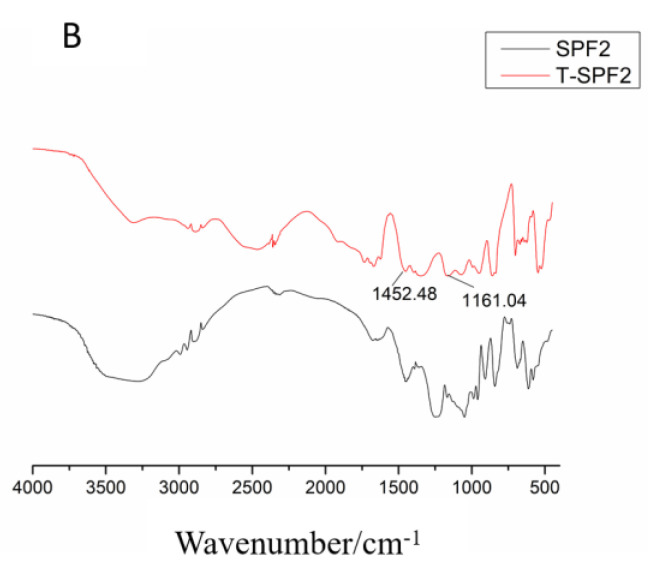
FT-IR spectra of F, T-F, SPF2, and T-SPF2. ((**A**) F, T-F, and (**B**) PF2, T-SPF2).

The spectra showed that T-F and T-SPF2 exhibited different absorption intensities for their functional groups, but all had characteristic peaks of sugar. This finding indicated that the reaction did not affect the main backbone of the sugar. Additionally, there was a C-S bond around 1200 cm^−1^ and an aromatic characteristic peak around 1500 cm^−1^ [40] in the Figure 9, which suggested that FITC was successfully connected to the sugar chain.

### 3.7. Quantitative Analysis of T-F and T-SPF2

#### 3.7.1. Standard Curve Plotting

A standard curve was plotted by using the mass concentration of plasma as the x-axis and fluorescence intensity as the y-axis. In the Table 2, the correlation coefficients of the linear regression equations for each sample were all greater than 0.99. This indicated that the linear equations meet the requirements for the determination of biological samples.

#### 3.7.2. Recovery, Precision, and Stability

In the Table 3. The recovery rates for T-F and T-SPF2 ranged from 93.62% to 102.43% and 93.49% to 99.61%. The relative standard deviations (RSD) were between 5.53%~10.76% and 4.34%~10.83%. The recovery rates of the labeled sugars were all greater than 90%. These results indicated that this method had good recovery.

The intra-day precision for T-F and T-SPF2 ranged from 4.14% to 10.20% and 4.34% to 10.83%. The inter-day precision ranged from 1.74% to 5.96% and 1.38% to 2.32%. Except for 1 μg/mL T-F and T-SPF2, the precision for the other concentrations was within 10%. These results indicated that this method had good precision.

The stability of T-F and T-SPF2 after 24 h at room temperature ranged from 4.04% to 5.56% and 2.74% to 9.21%. The stability after 24 h of refrigeration was between 3.00%~6.49% and 1.71%~9.45%. The stability after three freeze-thaw cycles ranged from 3.90% to 7.63% and 4.26% to 8.24%. The stability of the samples under different storage conditions was all within 10%. These results indicated that the stability of the samples was good.

### 3.8. Metabolic Kinetics Study in Mice

**Figure 10:**
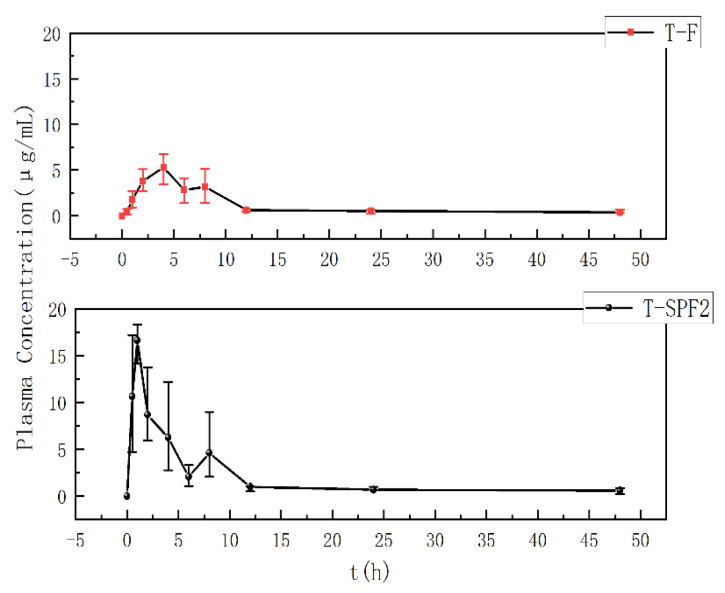
Plasma concentration-time profile of T-F and T-SPF2 after oral administration.

Pharmacokinetics primarily studied the absorption, distribution, metabolism, and excretion of substances within the body. T-F and T-SPF2 could all be absorbed into the bloodstream through oral administration. As shown in Figure 10, T-F could be detected in plasma with a fluorescence intensity of 0.5 h after gavage. This indicated that the body had begun to absorb T-F, which reached its peak at 4 h, followed by a decline, and then rose again at 8 h. This was related to the enterohepatic circulation of T-F, consistent with the results by Xu et al. [36]. T-F was absorbed into the bloodstream, which led to a gradual increase in blood drug concentration. And it was followed by a decline. Then T-F is absorbed in the liver, spleen, lungs, and kidneys, with the blood drug concentration increasing along with the enterohepatic circulation. These further demonstrated that high-molecular-weight fucoidan was absorbed in the human body [41]. But the high-molecular-weight fucoidan had a longer retention time and lower bioavailability. T-SPF2 showed detectable fluorescence intensity in plasma 0.5 h after gavage, with a blood concentration reaching 10.63 μg/mL, and it peaked at 1 h. T-SPF2 reached its peak concentration faster than T-F at a higher maximum concentration. This finding indicated that lower molecular weight substances had better absorption efficacy.

The blood concentration data of T-F and T-SPF2 were used to conduct pharmacokinetic simulations by using DAS 2.0 in mice; the pharmacokinetic parameters are shown in Table 4. The area under the curve (AUC_0–∞_) for T-F and T-SPF was 73.03 mg/L h and 375.95 mg/L h. The mean residence time (MRT_0–∞_) for T-F and T-SPF2 was 51.03 h and 17.95 h. The absorption of T-SPF2 was five times higher than that of the same dose of polysaccharide. While the distribution and elimination processes of oligosaccharides were slower. The time required for elimination from the body was shorter. It seemed that fucoidan oligosaccharides had a higher area under the curve (AUC_0−*t*_) and were absorbed more quickly than fucoidan. These results are consistent with the results by Jiaojiao T et al. [42]. Jiaojiao T’s experiment indicated that the bioavailability of low-molecular-weight fucoidan is 28.3%, while E. Z’s experiment showed that the bioavailability of fucoidan is 8.91% [43]. Therefore, it could be concluded that the bioavailability of fucoidan was low. This series of pharmacokinetic parameters indicated that oligosaccharides were rapidly absorbed and had higher bioavailability, which was related to their molecular weight.

## 4. Discussion and Conclusions

Fucoidan was a polymer formed by α-L-fucose-4-sulfate esters linked by connections at C1, C2; C1, C3; and C1, C4. Fucoidan from these sources had a complex molecular structure, which primarily consisted of two basic structures; one was predominantly linked by (1→3)-α-L-fucopyranose, and the other consisted of alternating (1→3) and (1→4) linked α-L-fucopyranose. The current research indicated that the structure after acid hydrolysis was similar to fucoidan, which was a sulfated oligosaccharide, consistent with the results of this study [44]. In this study, fucoidan oligosaccharides primarily consisted of Fuc_3_S_3_, Fuc_4_S_4_, and Fuc_2_S_2_, with Fuc_3_S_3_ being the most abundant. These findings indicated that fucoidan oligosaccharides obtained after acid hydrolysis and gel column separation purification consist of a mixture of sulfated oligosaccharides with the same composition and structure, rather than just repeating tetrasaccharide units [45].

Caco-2 cells are derived from human colon cancer cells, which could exhibit characteristics of intestinal epithelial cell differentiation when cultured on porous membranes in vitro. Active transport systems in Caco-2 cells showed similar expression patterns. Caco-2 cells could reveal the transport mechanisms of nutrients in vivo and the pathways of intestinal absorption by the transport time, the excretion time, the transport rate, and the apparent permeability coefficients [46,47]. In this study, F, SPF1, and SPF2 could be absorbed by small intestinal epithelial cells, and the uptake rate on the apical (AP) side was significantly higher than that on the basal (BL) side. The high-molecular-weight crude sugar F could be absorbed by the intestine, but it is similar to poorly absorbable substances. Both fucoidan oligosaccharides SPF1 and SPF2 were classified as moderately absorbable substances, but SPF2 is absorbed better than SPF1. The transport and uptake of fucoidan oligosaccharides in Caco-2 cells increased with time. The lower concentrations achieved better transport and uptake effects [48]. These results indicated that the transport mechanism of fucoidan oligosaccharides might involve active transport that required a carrier. And the smaller molecular weight facilitated absorption and transport by cells.

Fucoidan oligosaccharides exhibited good biological activities, which included antioxidant, anti-inflammatory, anticancer, and immune response effects [49]. Oligosaccharides’ tissue distribution and pharmacokinetics had become research hotspots because of low molecular weight, toxicity, and good bioavailability. Pharmacokinetic studies often utilized rat and mouse experiments to explore aspects such as absorption, distribution, and metabolism of substances. T-F and T-SPF2 were administered to mice by oral gavage. Blood samples were collected at different time points by tail clipping to observe the absorption in the mice. The results showed that fucoidan oligosaccharides were absorbed into the bloodstream more quickly, with a rapid absorption rate and short elimination time. Low-molecular-weight fucoidan exhibited better absorption properties than high-molecular-weight variants, which allowed for faster distribution to various tissues after entering the bloodstream and accumulated in the kidneys and liver [36]. These indicated that oligosaccharides were more easily absorbed by the body compared to polysaccharides.

In this study, fucoidan was degraded by acid and separated by Bio-Gel P10 to obtain two fucoidan oligosaccharides with different molecular weights, named SPF1 and SPF2. The absorption patterns of these oligosaccharides were further studied in vitro and in vivo, which used a Caco-2 cell monolayer model and animal experiments. The results were consistent with the study by Wang [50]. These indicated that fucoidan oligosaccharides could be transported more rapidly than fucoidan through the Caco-2 cell monolayer model, with smaller molecular weights, which exhibited better transport efficiency. The transport depended on relevant carriers. F was similar to difficult-to-absorb substances, while SPF1 and SPF2 were classified as medium-absorbable substances. The ratio of the *P_app_* value on the AP side to the BL side was greater than 1.5. This indicated that the transport mode was active transport, with the absorption effect being greater than the efflux effect. This study provided a foundation for understanding the absorption mechanisms of fucoidan oligosaccharides in vivo, although the specific transport modes required further investigation.

Fucoidan oligosaccharides were degradation products of fucoidan, with a smaller molecular weight and simpler structure compared to polysaccharides. The oligosaccharides possessed higher absorption efficiency. The results indicated that fucoidan oligosaccharides had a more pronounced regulatory effect on the intestines and provided essential nutrients. This study explored the absorption patterns of fucoidan oligosaccharides in Caco-2 cell models and in mice, providing a theoretical basis for further research on fucoidan oligosaccharides.

## Figures and Tables

**Table 1:** Chemical constituents of F, SPF1, and SPF2.

Sample	F	SPF1	SPF2
Total sugar (%)	51.90 ± 1.21 ^c^	68.67 ± 2.90 ^a^	59.70 ± 0.97 ^b^
Sulfate group (%)	25.58 ± 1.02 ^a^	15.33 ± 0.41 ^c^	16.89 ± 0.50 ^b^
Aldonic acid (%)	-	2.15 ± 0.35	-
Range of molecular weight (Da)	-	1.63 × 10^4^~2.14 × 10^5^	244.22~1545.36
Composition of monosaccharides (%)			
Fucose	83.97	12.76	97.94
Xylose	0.59	5.00	0.28
Rhamnose	0.57	5.36	0.25
Mannose	6.47	38.87	0.19
Galactose	2.76	8.67	0.81
Glucuronic acid	3.37	25.59	0.17
Galacturonic acid	-	0.07	-
Glucose	2.27	3.69	0.35

**Note:** Differences with different letters in the same row are significant (*p* < 0.05), while those with the same letter are not significant (*p* > 0.05).

**Table 2:** The linear regression equation and the correlation coefficients of biological samples.

	Linear Equation	R^2^	Linear Range
T-F	y = 30.005x + 0.9579	0.9991	1~25 μg/mL
T-SPF2	y = 27.37x + 4.0118	0.9994	1~25 μg/mL

**Table 3:** Recovery, precision, and stability of T-F and T-SPF2 in mouse plasma (*n* = 5).

Sample	Concentration (μg/mL)	Recovery	Precision		Stability		
		RSD (%)	RSD (%) Intra-day	RSD (%) Inter-day	RSD (%) Room Temperature for 24 h	RSD (%) Refrigerate for 24 h	RSD (%) Re-Dissolve Three Times
T-F	1	10.76	10.2	3.75	5.56	6.49	7.63
	5	10.52	4.14	5.96	4.04	6.02	6.24
	25	5.53	5.12	1.74	4.66	3.00	3.90
T-SPF2	1	10.83	10.83	1.38	9.21	9.00	8.24
	5	6.17	6.17	2.32	6.10	9.45	7.33
	25	4.34	4.34	1.98	2.74	1.71	4.26

**Table 4:** Pharmacokinetic parameters of T-F and T-SPF2 after oral administration.

Sample	C_max_	T_max_	T_1/2_	AUC_0–t_	AUC_0–∞_	MRT_0–∞_	CL/F
	(mg/L)	(h)	(h)	(mg/L h)	(mg/L h)	(h)	(L/h/kg)
T-F	5.28	4.00	51.12	43.79	73.03	51.03	3.10
T-SPF2	16.63	1.00	12.11	274.02	375.95	17.95	0.84

**Note:** C_max_, peak concentration; T_max_, time to peak concentration; T_1/2_, half-life; AUC_0–t_, area under the curve from zero to t; AUC_0–∞_, area under the curve from zero to infinity; MRT_0–∞_, mean residence time; CL/F, ratio of plasma clearance to absolute bioavailability.

## Data Availability

The original contributions presented in this study are included in the article; further inquiries can be directed to the corresponding author.
